# Detection of Gallbladder Disease Types Using Deep Learning: An Informative Medical Method

**DOI:** 10.3390/diagnostics13101744

**Published:** 2023-05-15

**Authors:** Ahmed Mahdi Obaid, Amina Turki, Hatem Bellaaj, Mohamed Ksantini, Abdulla AlTaee, Alaa Alaerjan

**Affiliations:** 1 CEMLab, National School of Electronics and Telecommunications of Sfax, University of Sfax, Sfax 3029, Tunisia; 2 CEMLab, National Engineering School of Sfax, University of Sfax, Sfax 3029, Tunisia; amina.turki@enis.tn (A.T.); mohamed.ksontini@enis.rnu.tn (M.K.); 3 ReDCAD, National Engineering School of Sfax, University of Sfax, Sfax 3029, Tunisia; hatem.bellaaj@redcad.org; 4 Croydon Hospital, London CR7 7YE, UK; abdullahsafaaldin@gmail.com; 5 College of Computer and Information Sciences, Jouf University, Sakaka 72388, Saudi Arabia; asalaerjan@ju.edu.sa

**Keywords:** artificial intelligence, deep learning, deep neural network, ultrasound images, diagnosis, gallbladder

## Abstract

Nowadays, despite all the conducted research and the provided efforts in advancing the healthcare sector, there is a strong need to rapidly and efficiently diagnose various diseases. The complexity of some disease mechanisms on one side and the dramatic life-saving potential on the other side raise big challenges for the development of tools for the early detection and diagnosis of diseases. Deep learning (DL), an area of artificial intelligence (AI), can be an informative medical tomography method that can aid in the early diagnosis of gallbladder (GB) disease based on ultrasound images (UI). Many researchers considered the classification of only one disease of the GB. In this work, we successfully managed to apply a deep neural network (DNN)-based classification model to a rich built database in order to detect nine diseases at once and to determine the type of disease using UI. In the first step, we built a balanced database composed of 10,692 UI of the GB organ from 1782 patients. These images were carefully collected from three hospitals over roughly three years and then classified by professionals. In the second step, we preprocessed and enhanced the dataset images in order to achieve the segmentation step. Finally, we applied and then compared four DNN models to analyze and classify these images in order to detect nine GB disease types. All the models produced good results in detecting GB diseases; the best was the MobileNet model, with an accuracy of 98.35%.

## 1. Introduction

The gallbladder (GB) is a tiny pouch and a hollow organ located beneath the liver. Its primary role is to temporarily store bile. Bile is a fluid formed by the liver, which is used to aid digestion. There are different types of GB disease. From gallstones to cancer, they all have similar symptoms, but they vary widely in severity [1]. All scientists confirm that although the exact causes of GB cancer are unknown, certain factors may increase a person’s risk of developing GB cancer. Frequently, these factors are related to the simple inflammation of the GB. According to the American Cancer Society (ACS) [2] and the American Society of Clinical Oncology (ASCO) [3], the risk of GB cancer is around five times higher in people who have a history of GB conditions, mainly gallstones, compared to those who do not. To this end, it is crucial to diagnose the type of GB disease and how serious it is at an early stage in order to prevent or reduce the wider spread of the disease.

Early detection and diagnosis are the primary sources of lifesaving and are among the most-challenging features of health surveillance. Indeed, according to the Canadian Cancer Society (CCS), about 19% of people diagnosed with GB cancer survive for at least 5 years. Unfortunately, only about 4% of those with stage 4 survive their cancer for 5 or more years, compared to 50% of those diagnosed with stage 1.

To perfectly diagnose GB diseases, ultrasound imaging, as one of the most frequently used imaging modalities, is recognized as a powerful and universal screening and diagnostic tool for physicians and radiologists [4]. After screening, an accurate diagnosis is necessary to identify an appropriate disease treatment plan. Typically, diagnostic information is collected from the patient’s history and clinical examination. Many indications and symptoms can be ambiguous, especially those related to GB diseases, which have similar symptoms. Therefore, ultrasound images (UI) have to be interpreted and understood by highly qualified medical professionals. Given that diagnosis via ultrasound imaging can be time- and labor-consuming, it can be difficult to fund and take advantage of this service in remote locations [5]. Regretfully, many analysts refuse to work in rural areas, and some hospitals do not have the resources to train existing medical professionals to provide this service. As a result, an informative method is needed to simplify the UI acquisition and evaluation process in order to recognize the organ-related pathologies and anomalies in a widely accessible and achievable manner. In rural locations, this informative method can be very useful in computerized healthcare structures, which is the prime motivation of the proposed study. Indeed, if any anomalies are discovered during the initial screening step, specialists and radiologists can detect intraabdominal organ issues and provide the exact treatment. Unskilled radiologists can also use the produced healthcare model to build relative research on scans for evaluating different solutions [6].

Currently, artificial intelligence (AI) techniques, ranging from machine learning (ML) to deep learning (DL), are prevalent in healthcare for disease diagnosis, drug discovery, drug development, and patient risk identification [7]. The advances in DL and deep neural network (DNN)-based methods of research and development provide significant progress in the domain of medical image analysis and understanding [8]. Moreover, with the progress at the algorithmic level as well as the availability of high-performance computing machines and large quantities of data, DL-based methods have become increasingly popular [9]. They are now considered to be the most commonly used and most sophisticated algorithms for handling many computer vision tasks [10]. In addition, DL algorithms are capable of assisting analysts in the early identification, treatment, and recognition of diseases, and they, subsequently, provide efficient methods for medical diagnostics. Indeed, DL algorithms can directly process and automatically learn mid-level and high-level abstract features acquired from immense quantities of raw collected data, in which higher-level abstract features are defined by combining them with lower-level features, to achieve an acceptable level of accuracy and, eventually, to perform automatic UI analysis tasks, such as classification, organ segmentation, and object detection [9,11,12,13,14].

Few studies have used DL to detect GB-related conditions, despite the fact that some of them used DL to treat various GB-related conditions [15]. In this paper, we propose an informative medical method based on four DNN models for detecting intra-abdominal organs as well as associated diseases using UI. This method can be used to detect nine GB diseases, including cancer, at the same time, based on a built dataset. The used dataset was carefully gathered from a wide variety of sources over three years. It consists of more than 10,000 UI of the gastrointestinal tract from 1782 patients. After preprocessing the data, we applied DL models, including VGG16, InceptionV3, ResNet152, and MobileNet, to recognize the GB organ and the nine associated diseases. This technique has the potential to be a game changer in the medical field, especially for radiologists and other clinicians who deal with patient care.

In Section 2 of this paper, we briefly introduce and discuss a variety of GB pathologies. Section 3 comprises a literature review of the existing research related to this topic. In Section 4, the description of the datasets is presented. In Section 5, the implementation of the proposed informative method is discussed. In Section 6, an exploration of the results of the proposed approach is discussed and concluded. Section 7 presents the conclusion and future works.

## 2. Overview of GB Diseases

The GB is located on the lower surface of the right side of the liver and has separate anatomic sections, including the neck, fundus, cystic duct, and infundibulum. The GB is divided into four layers: (1) the mucosa, (2) the muscularis, (3) the perimuscular layer, and (4) the serosa. The GB is also complicated, despite its small size [16]. In total, 10–15% of the adult population is affected by GB disorders, so it is comparably prevalent, with the most common pathology being cholelithiasis [17]. Next, we provide a summary of some of the diseases that affect the GB.

### 2.1. Gallstones

The components that make up bile are numerous, with the most important being cholesterol, bilirubin (a by-product of red blood cell degradation), and bile salts, all of which are dissolved in water. These components are often produced by the liver and stored in the GB.

**Figure 1:**
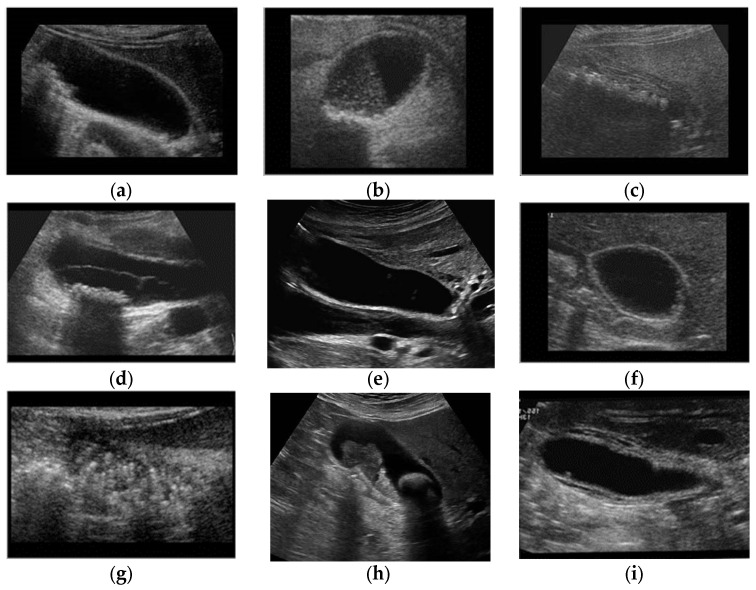

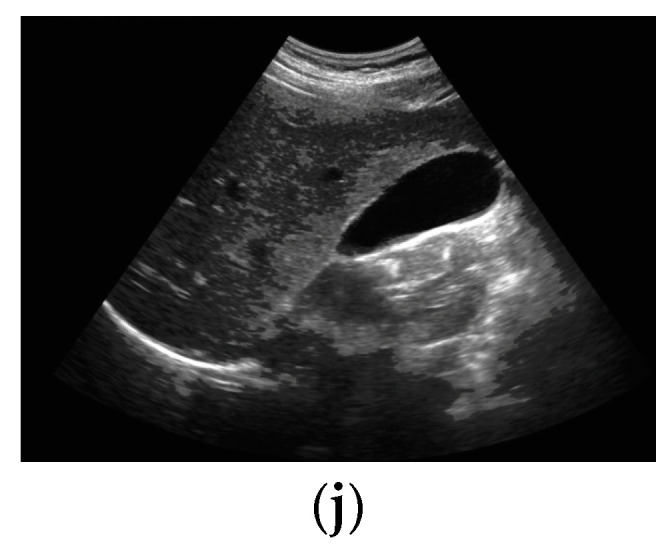
GB diseases (**a**–**i**): (**a**) gallstones, (**b**) abdomen and retroperitoneum, (**c**) cholecystitis, (**d**) gangrenous cholecystitis, (**e**) perforation, (**f**) polyps and cholesterol crystals, (**g**) adenomyomat osis, (**h**) carcinoma, (**i**) GB wall thickening, and (**j**) normal GB.

Gallstones form as a result of an imbalance in those components, for example, increased cholesterol (due to high liver output) forms cholesterol gallstones in the GB (the most common form) (Figure 1a). The exact cause of these derangements is multifactorial and is not the purpose of this paper. Gallstones can remain asymptomatic until the duct that transports bile into the small intestine becomes blocked by a gallstone, which induces a variety of symptoms depending on the anatomical location and severity of the blockage. This often necessitates treatment, which can be in the form of elective surgical removal of the stone and GB [18].

### 2.2. Biliary Colic and Calculous Cholecystitis

Temporary pain due to the obstruction of the cystic duct by a gallstone upon bile secretion is known as biliary colic. This pain usually quickly subsides, but, if the obstruction persists, it can lead to cholecystitis (as shown in Figure 1c) [19,20]. Prolonged blockage of the cystic duct can cause inflammation in the GB, leading to GB wall thickening (Figure 1i). This inflammation can result in fever, lethargy, and constant pain.

### 2.3. Gangrenous Cholecystitis

Cholecystitis that remains untreated eventually causes the GB to turn gangrenous (Figure 1d) [21]. This occurs as a result of edema, which induces vascular insufficiency to the GB, leading to ischemia of the GB tissue. It is a life-threatening condition due to the high risk of perforation (Figure 1e) [22], which may lead to hemodynamic instability [23,24].

### 2.4. Polyps and Cholesterol Polyps

Polyps are uncommon, with a 9–26% frequency range based on surgery and autopsy sequencing data. The most common are cholesterol polyps. They occur as a result of a buildup of lipid within the macrophages in the lamina propria projecting into the inner lumen of the GB; this process is known as cholesterolosis. These are benign growths (Figure 1f), as they are made from cholesterol deposition rather than neoplastic growths [25].

### 2.5. Adenomyomatosis of the GB

Adenomyomatosis of the GB is an illness characterized by aberrant mucosal epithelial hypertrophy, resulting in the pathognomonic epithelial invaginations known as Luschka’s crypts in the GB [26]. These crypts often house cholesterol crystals that aid in forming a distinct appearance upon imaging (Figure 1h). They have a frequency rate of between 1% and 9% in cholecystectomy specimens, with a steady sex proportion. They become more common after the age of 50, supporting the hypothesis that chronic inflammation is a cause.

### 2.6. Carcinoma

GB cancer [27] is a very rare tumor occurring in 1:100,000 of cases, mainly in those aged over 70. It is more common in females than males (2:1). Chronic inflammation is the most important risk factor for developing carcinoma, which is why it occurs in those with a history of gallstones (Figure 1g) [28,29] Carcinomas can metastasize to other parts of the body or remain confined to a specific area [30].

## 3. Literature Review

Early detection is one of the most important secondary prevention strategies for diseases. Secondary prevention includes early diagnosis and prevention, which allows medical staff to provide the required care for patients and improve their quality of life.

Researchers might employ modern technologies, especially AI approaches, to help detect diseases before they reach their late stages. To this end, several studies using AI techniques for ultrasound imaging were proposed. For example, Zhang et al. [31] constructed a convolutional neural network (CNN) model that captures UI of fatty livers using the scan’s gray and texture properties for sorting and classifies these UI. The results attained an accuracy of 90%, a specificity of 92%, and a sensitivity of over 81%. Zheng et al. [32] used deep transfer learning (DTL) approaches for the systematic organization of the kidneys. They identified children with genetic disorders of the kidneys and urinary tract from UI of the kidneys, based on the transfer learning approach and imaging features. They achieved an area under the curve (AUC) greater than 0.88. Arora and Mittal [33] proposed image enhancement techniques for gastric disease detection using UI. They used three types of filtering techniques, which are unsharp, wiener, and middle filters, to improve the ultrasound scans. The authors claimed that the best visible qualities were obtained with unsharp filtering. Selvan and John [34] described the use of form and texture functions to recognize aberrant masses in UI. Radhakrishnan and Raghesh Krishnan [35] proposed UI with wavelets and texture cues to classify focal and distributed liver diseases in a hybrid manner. They established a technique based on computers to identify 10 different types of localized and disseminated liver disorders. The unhealthy area of the UI was separated using the active contour segmentation (ACS) approach, with an overall accuracy of 91%. Acharya et al. [36] developed a model that uses a script for filter series and the structures of local outline forms for the detection of breast incisions in UI. The proposed model was able to accurately detect and classify breast lesions with 96.1% accuracy, 96.5% sensitivity, 95.3% specificity, and 97.9% positive predictive values.

Later on, many researchers used DL techniques for the same purpose. In fact, Liu et al. [37] discussed, in their review, the use of DL algorithms in ultrasound scan inspections for a variety of functions, such as recognition, segmentation, and classification. Similarly, Kumar and Bindu [38] conducted a review of image analyses by utilizing DL approaches. Sloun et al. [39] also discussed the use of DL in health ultrasound scanning approaches. Chen et al. [40] considered a deep CNN for biomedical scan methods in dentistry and medicine.

As previously mentioned, many diseases may affect the GB. A well-developed AI approach based on ultrasound GB images could increase illness detection accuracy. Urman et al. [41] studied the bile canaliculi and bile ducts in the gallbladder using a machine learning technique. Yao et al. [42] used a DL model to recognize gallstones while using massive amounts of data from the Internet of things (IoT). They also created a CNN for the acquired imaging records’ functioning features. Chang et al. [43] considered the utility of a backpropagation neural network and genetic algorithm in the detection and prediction of tumors’ signs in gallbladder cancer patients.

Ultrasound GB images represent one of the most-common images used for the detection of biliary Artesia (BA). In fact, Zhou et al. [44] developed a deep learning approach for BA evaluation. The model gave a specificity of 93.9% and a sensitivity of 93.1% at the patient level. Obaid et al. [45] used deep learning approaches and ultrasound GB images to detect BA, with specificities and sensitivities of more than 90%. Furthermore, a radiologist may be able to spot a pulmonary nodule on a chest X-ray [46], decipher an MRI of the knee [47], and spot a brain aneurysm on a magnetic resonance angiography based on a DL model [48]. Unfortunately, we were unable to find any examples in the literature that used DL to distinguish between GB diseases based on UI. Our research aims to ascertain if a DL can aid in the differential diagnosis of GB polyps and other diagnoses using ultrasound imaging. Therefore, this study recommends a new, precise, and trustworthy detection system for GB diseases. In order to identify abdominal organs from ultrasound scans utilizing NN and opacity, a sizable training setup is required, which can lead to the NN performing erroneously. The natural recognition of abdominal organs is growing increasingly difficult as a result of the inadequate quantity of findings, the disparity in media impact, the increasing differences in organ forms and locations, and the gray-level links of neighboring organs. Ultrasonic scans level the organ’s margins, since image denoising filters are used, making it difficult to distinguish between the shapes of different organs. The proposed method addresses these issues by utilizing systematic techniques at various working levels for intra-abdominal organ diagnosis. In contrast to systems utilizing local mean algorithms (LMA), our approach enhances the UI using a non-local mean (NLM) filter and a bilateral filter before segmentation, enabling the model to recognize the GB with greater efficiency and accuracy. The NLM filter efficiently brings out the key details in the image, making it well suited to segmentation.

## 4. Proposed Informative Medical Method

### 4.1. Contribution

The idea consists of the detection of nine GB diseases at the same time using four DNN models, based on a large dataset of medical images. This approach may represent an ambitious method to detect even fatal diseases at early stages.

### 4.2. Datasets

The dataset is composed of ultrasound images of the gallbladder organ from inside the gastrointestinal tract. It was collected from three hospitals, Al-Nahman Teaching Hospital, Medical City Hospital, and Jenin Al-Ahly Hospital, as well as from two centers, Al-Amal Center and the Gastroenterology Center in Baghdad. The collection of data was collaboratively undertaken, under the supervision of a specialist in gastroenterology. After collection, these images were carefully sorted by a specialist in radiology at the Gastroenterology Center of the Medical City Hospital to obtain, at the end, 10,692 useful ultrasound images. The obtained images were then classified by the same team into nine classes of gallbladder-related diseases, according to the pathological findings. Each class provides nearly 1200 images. Therefore, the dataset is balanced in terms of diseases. In total, 782 patients were involved in the data collection; the number of female images was 6246, with an average age of 63.4, while the number of male images was 4446, with an average age of 59.6. Furthermore, 80% of the dataset (8553 images) were used for the training step, and 20% (2139 images) were used for the testing step. When constructing a train/test split for a dataset that includes multiple images from the same patient, it is important to ensure that all images from a particular patient are placed in either the training or testing set but not both. This is because if the images from the same patient are included in both the training and testing sets, it can lead to overly optimistic results, as the model may simply memorize features of a particular patient rather than learning more generalizable patterns. To achieve this, one common approach is to group the images by patient ID and then randomly assign patients to either the training or testing set, such that all images from a given patient are placed in the same set. The following conditions were considered for the image processing:

1. The initial values were divided by 255, and the intensity value for each pixel was rescaled into the range of (0, 1).
2. Arbitrary zooming and shearing were performed in order to make the model more robust with slight changes in inputs.
3. All images were horizontally flipped.

### 4.3. Implementation

Most of the previous works considered the classification of only one disease of the GB, but, in our work, we focus on detecting nine diseases at once and on determining the type of disease using UI. However, in this study, we develop a novel conceptual model for detecting intra-abdominal organs using ultrasound imaging. The implementation of this study is based on three steps:

**Step 1**: Enhancement of UI.
**Step 2**: Region of Interest (ROI)-based image segmentation with the help of DNN.
**Step 3**: Identification of intra-abdominal organs using four DNN algorithms.

**Figure 2:**
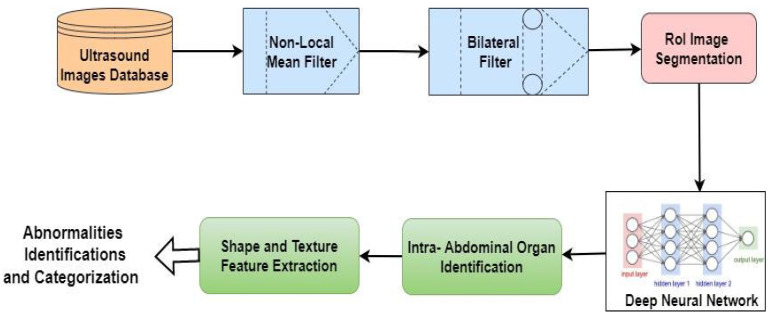
Flowchart representing the working of GB disease detection approaches.

Several researchers used DL techniques to detect intra-abdominal organs, such as the kidneys [49], breasts [50], pancreas [51], stomach [52], liver [53], and others. Moreover, a DL algorithm is used to recognize the GB disease. Following the identification of this organ, an objective technique for UI is used to split the additional ROI. The patients’ personal information is not included in the record. The images are degraded by speckle noise and Gaussian noise throughout the ultrasonic scan. Through the denoising of scans, it is possible to improve the quality of UI. The scan endpoints are preserved using a bilateral filter and an NLM filter. Nearly 20% of the data are used as a testing set for the DL algorithms. The subdivisions below provide a detailed description of each stage of the established approach. The flowchart of the implementation is shown in Figure 2. The role of the segmentation task is then to determine the meaning of each pixel in an image and label it accordingly. In order to simplify an image and analyze it more effectively, it is often separated into different areas based on the properties of the pixels that indicate objects or borders.

#### 4.3.1. Step 1: Enhancement of Ultrasound Images

To enhance the ultrasound images, a bilateral filter and an NLM filter were applied to the initially processed UI in order to detect anomalies in the intra-abdominal organs. The use of NLM filtering restores the majority of pixels in an image, based on their similarity to the target pixel. Compared to LMA, the use of NLM filtering can guarantee less feature loss and significantly more post-filtering clarity. A bilateral filter adds a second layer of filtering to protect the organ’s boundaries. A bilateral filter modifies the power of each pixel with a weighted mean of the power values from neighboring pixels. Finally, better UI were used to divide the ROI of the organ. To increase the quality of the UI, it is necessary to recall the element data in the image and safeguard the edges.

#### 4.3.2. Step 2: Region of Interest (ROI)-Based Image Segmentation

The u-net architecture was widely used in cell or tissue segmentation and achieved very good performance on very different biomedical segmentation applications [54]. It is especially effective with limited dataset images. In this work, we used the active contour method for segmentation to extract the region of interest (ROI) from medical images of the GB, followed by classification using a deep neural network (DNN) to identify the type of GB disease. Actually, it is common practice for medical scans to have “regions of interest” (ROI). The primary goal of organ and disease detection in an ultrasonic image analysis is to pinpoint the ROI, so it can be used as a reference point during the segmentation process. The ROI contains crucial diagnostic data that can be used for pathological examination and subsequent clinical treatment. The primary goal of segmentation is, in fact, to enhance the readability and significance of medical images. Thus, in order to determine what part of the body has to be looked at, it is important to segment the medical image and extract the ROI. Many rounds of morphological assessment are built into the standard segmentation method. Therefore, we utilized the same DNN that was used for classification to automatically segment the UI in order to localize any ROIs that contain the organ. The first step was to segment the medical images of the GB in order to extract the region of interest (ROI). This was accomplished using the active contour method. The active contour method, also known as snakes, is a technique used for detecting and delineating boundaries in an image. It is a type of energy minimization method that relies on the curve evolution to identify the boundaries. The method uses a curve that deforms over iterations to align with the edges of the object in the image. The curve is attracted to the boundaries by the internal energy of the curve, while it is repelled from other features in the image by the external energy.

**Figure 3:**
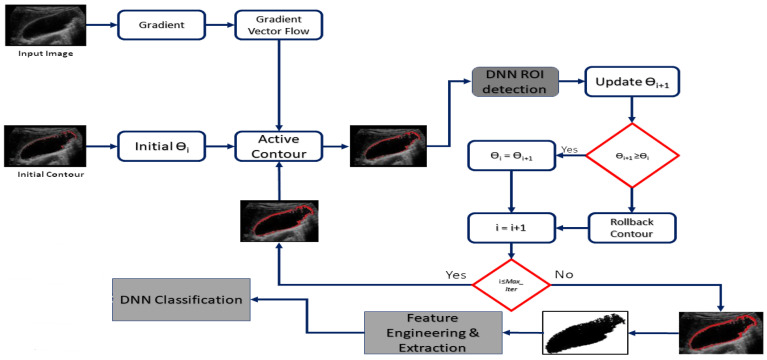
Workflow for using the DNN for segmentation and classification.

In this process, the active contour was initiated with a seed point, and the curve evolved until it reached the boundary of the object of interest. During the evolution of the curve, a deep neural network (DNN) was used to determine whether the masked region is an interested part, to allow the active contour to continue and find out the complete ROI. Each increment in the elected ROI was introduced as an input for the DNN, which yielded a detection score of recognition. The score was positively relative to the context of the elected ROI, which increased or decreased as an indicator of the correctness of the active contour’s progress. Each increment of the recognition score caused the active contour to progress further, and vice versa. For an invalid progress of the active contour, the ROI removed the recently added section and tried to increase other sections until it reached the maximum iteration and obtained the final ROI. The DNN model is used for classification and diagnosis the disease. Figure 3 represents a complete workflow for using the DNN twice, to utilize the segmentation process and improve the classification accuracy.

#### 4.3.3. Step 3: Identification of GB Disease Using Four DNNs

**Figure 4:**
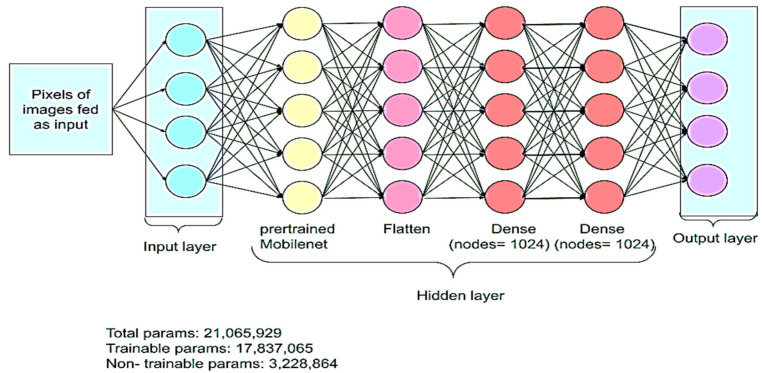
Model architecture.

We applied many DNNs to the segmented images in order to identify the GB disease type. To accomplish this task, we used many transfer learning DNNs’ algorithms. The best results were detected by the following four deep CNN models: VGG16, InceptionV3, ResNet152, and MobileNet. Each model relied on a set of pre-trained weights obtained from ImageNet. We then added more deep layers to the models to make them more suitable for our purpose. As a first step, the pre-trained model’s output was passed to a flat layer, which flattened the multi-dimensional vector into a single-dimensional one. The subsequent dense layers took this vector as the input. We used two dense layers, each with 1024 neurons and the rectified linear unit (ReLU) activation function. This function was used to apply the required nonlinear transformations to transform the input at each node to the corresponding output. After these dense layers, we added a dropout layer with the dropout rate set to 0.5 to reduce overfitting and to enable the model to generalize well. Finally, the output from the dropout layer was used as the input for the last dense layer with nine neurons and the Softmax activation function, which was used to give the probability of the input image belonging to each class. The structure of the model used is shown in Figure 4.

During training, it is necessary to make frequent changes to the weights of the nodes in the network and to update these weights after each forward pass, so the gaps between the observed and predicted values are as small as possible. The backpropagation algorithm is used to do this task in a layer-by-layer fashion. For each iteration, the model’s performance is evaluated, and the weights are fine-tuned based on the magnitude of the propagated mistake. The model is obtained after 15 iterations using the Adam optimizer and a learning rate of 0.001.

## 5. Results

To evaluate the proposed approach, we trained the four previously described CNN models (VGG16, InceptionV3, ResNet152, and MobileNet) using our dataset. A total of 80% of the dataset was used for training, while 20% was used for testing. The dataset used in the training phase was different from the one used in the testing phase.

The performance metrics, which were used to measure the ability of the different models to detect GB diseases, are accuracy, sensitivity, specificity, F1 score, positive predicted value (PPV), negative predicted value (NPV), AUC, time processing, and confusion matrix. In the confusion matrix, true positive (TP) denotes a hazardous state that has been accurately detected, whereas true negative (TN) denotes a non-hazardous state that has been successfully identified. A non-hazardous state that is mistakenly identified as a hazardous state is referred to as a false positive (FP), while a precarious state that is mistakenly identified as a non-hazardous state is referred to as a false negative (FN). A confusion matrix can be used to determine the performance of a binary classifier.

As shown in Table 1 and Table 2, we deduced that all the models gave good results, while recognizing that MobileNet outperformed the other models when using the same dataset. Consequently, MobileNet was selected as the optimal model for this task, with an accuracy of 98.35%. The time processing became high as the number of layers increased, and it was relative to all the hardware and software materials.

**Figure 5:**
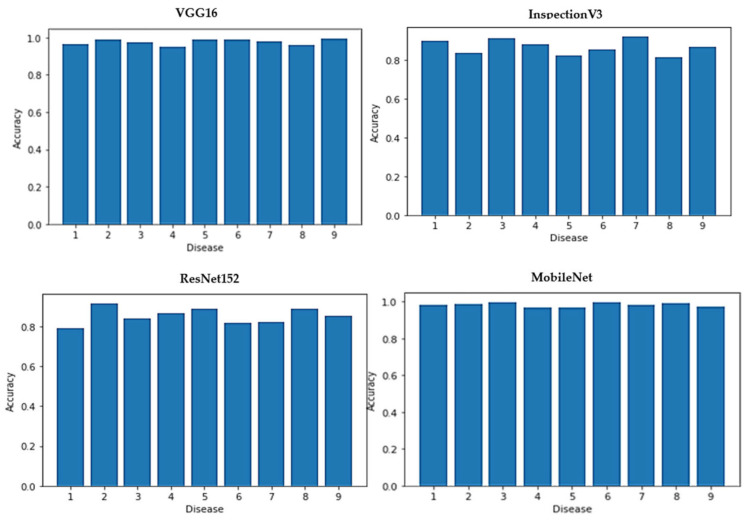
Accuracy of each disease for each model.

**Figure 6:**
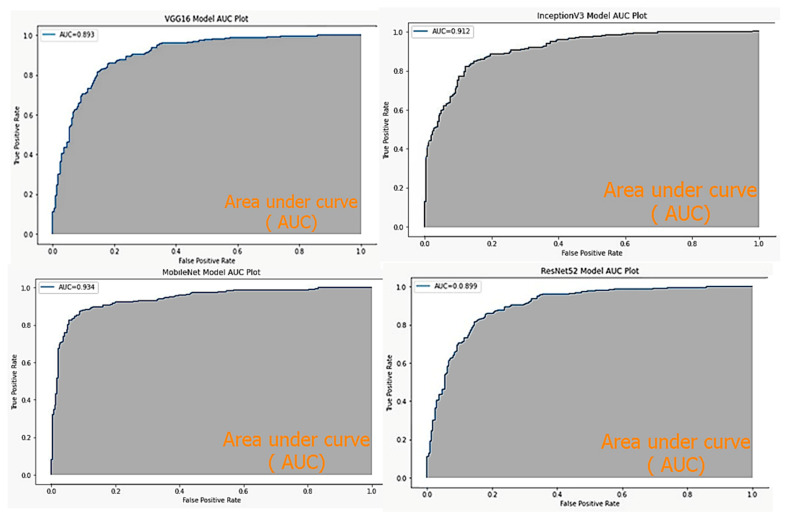
ROC curve and AUC value for each model.

Figure 5 and Figure 6 represent respectively the accuracy repartitions for each disease of each model and the ROC curve of each used model.

**Figure 7:**
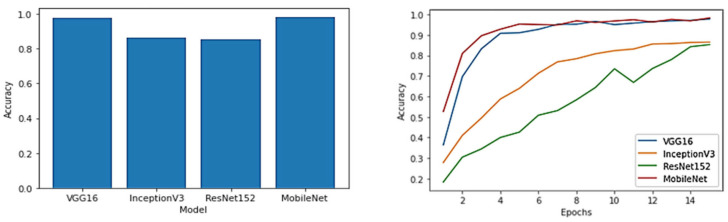
Accuracy and training history for each model.

Figure 7 compares the accuracy for each model.

**Figure 8:**
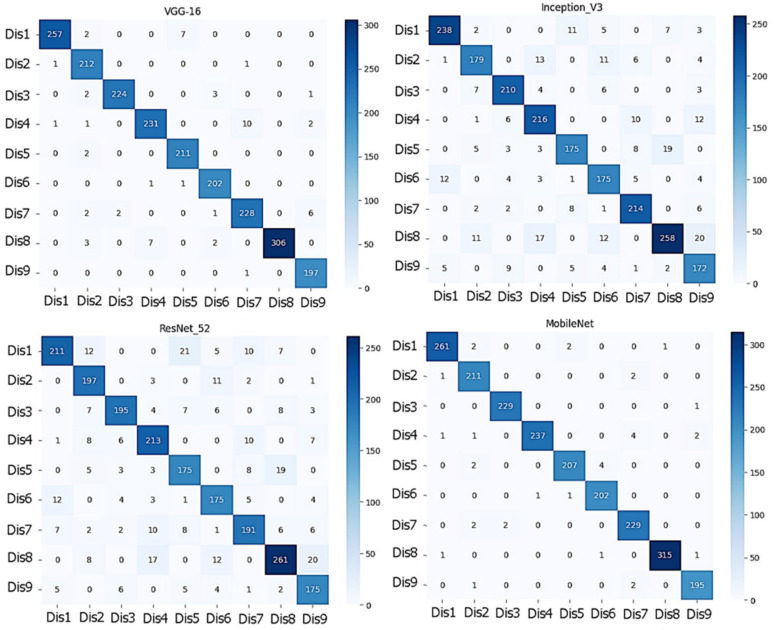
The confusion matrix for each model.

Figure 8 shows the confusion matrix for each model.

## 6. Discussion

According to the above findings, the VGG16, InceptionV3, ResNet152, and MobileNet models’ parameters were strong predictors of the GB-disease-type diagnosis, using UI as the datasets. Figure 7 shows that the proposed approach outperforms the benchmark models in terms of accuracy, when using the same number of epochs. With regard to the results of the different models, MobileNet represents the best model for detecting the different types of GB disease, with an accuracy of 98.35%. Other models were used, but the best results were produced when using the aforementioned models.

The proposed approach uses DNN to identify the organ from a set of intra-abdominal ultrasound pictures, while the shape and texture attributes are used to identify GB abnormalities. By gathering comprehensive data about a larger number of patients with various diseases proposed AI system can be developed and used for numerous other systems of the body and other diseases.

This study successfully joins several other pieces of research that proved the aptitude of deep learning in the detection of diseases. A variety of GB disease types were successfully identified, and adequate values for classifying a serious GB disease were obtained. Therefore, this approach represents a computer-aided diagnosis.

## 7. Conclusions

In the last few years, many studies have been conducted for AI research in the medical sector, to support experts in the early detection of diseases as well as in the prediction of certain syndromes. Usually, diagnosing GB diseases is difficult for specialists, especially beginners, so a diagnosis might be incorrect, leading to poor outcomes.

In this paper, we proposed an informative medical method using DNNs to detect the GB and its diseases by analyzing ultrasound scans of the organ. For this, a large dataset of intra-abdominal UI was carefully collected over three years.

To our knowledge, this is the first study using DNN to differentiate GB diseases. Thus, the novelty of this method is the simultaneous detection of this organ and nine different diseases affecting the GB at the same time, based on UI. In order to recognize the GB with greater efficiency, we used a non-local mean (NLM) filter and a bilateral filter on the UI before segmentation, enabling the model to recognize the GB with high accuracy and to make diagnosis more objective, accurate, and intelligent. Indeed, the suggested method used DL models, including the VGG16, InceptionV3, ResNet152, and MobileNet algorithms. MobileNet produced the best outcomes, with an accuracy rate of 98.35%. This approach has the potential to be a game changer in the medical field, especially for radiologists and other clinicians who deal with patient care.

However, the low contrast between the target and the background in the images and aberrations in ultrasound scanning could generate problems in intra-abdominal organ segmentation, making finding all the intra-abdominal organs a difficult task. It is essential to develop advanced automatic segmentation and ultrasound image analysis methods to overcome this issue. In addition, 3D ultrasound scanning can provide more robust results than 2D imaging.

Finally, our next investigations will focus on the difficult problem of detecting GB disease in ultrasound pictures utilizing mobile phone photographs, videos, and region-based convolutional neural network (R-CNN) technology.

## Figures and Tables

**Table 1:** The performance metrics for all models used for GB disease detection.

Metrics	VGG16	InceptionV3	ResNet152	MobileNet
Accuracy	0.9778	0.8650	0.8530	0.9835
Sensitivity	0.9974	0.8372	0.8213	0.9830
Specificity	0.9795	0.9882	0.9913	0.9979
PPV	0.9795	0.8984	0.9221	0.9838
NPV	0.9970	0.9798	0.9779	0.9978
F1 score	0.9779	0.8667	0.8688	0.9834
AUC	0.8930	0.9120	0.8990	0.9340
Time processing (s)	561	651	752	540

**Table 2:** The accuracies attained by each model for each GB disease.

Disease Number	Type of Disease	VGG16	InceptionV3	ResNet152	MobileNet
Dis1	Gallstone	0.965	0.8950	0.79	0.98
Dis2	Abdomen and retroperitoneum	0.9906	0.8350	0.9195	0.9870
Dis3	Cholecystitis	0.9746	0.91	0.84	0.998
Dis4	Gangrenous cholecystitis	0.9498	0.88	0.866	0.969
Dis5	Perforation	0.99	0.82	0.89	0.97
Dis6	Polyps and cholesterol crystals	0.99	0.85	0.816	0.997
Dis7	Adenomyomatosis	0.9778	0.92	0.82	0.9835
Dis8	Carcinoma	0.96	0.81	0.886	0.9935
Dis9	GB wall thickening	0.995	0.865	0.853	0.9835

## Data Availability

The datasets generated during the simulation study are available from the corresponding author on reasonable request.
